# Field evaluation of synthetic lure (3-methyl-1-butanol) when compared to non odor-baited control in capturing *Anopheles* mosquitoes in varying land-use sites in Madagascar

**DOI:** 10.1186/s13071-015-0729-1

**Published:** 2015-03-07

**Authors:** Sarah Zohdy, Kristin Derfus, Mbolatiana Tovo Andrianjafy, Patricia C Wright, Thomas R Gillespie

**Affiliations:** Department of Environmental Sciences and Program in Population Biology, Ecology, and Evolution, Emory University, 400 Dowman Drive, Suite E510, Atlanta, 30322, GA USA; Department of Environmental Health, Rollins School of Public Health, Emory University, 1518 Clifton Road NE, Atlanta, 30322, GA USA; Centre ValBio, BP 33 Ranomafana Ifanadiana 312, Ranomafana, Madagascar; Department of Entomology, University of Antananarivo, Antananarivo, Madagascar; Department of Anthropology, Stony Brook University, Stony Brook, 11794-4364, NY USA

**Keywords:** Malaria, Trap, Livestock, Ranomafana national park, Deforestation

## Abstract

**Background:**

Malaria is the 4^th^ largest cause of mortality in Madagascar. To better understand malaria transmission dynamics, it is crucial to map the distribution of the malaria vectors, mosquitoes belonging to the genus *Anopheles*. To do so, it is important to have a strong *Anopheles*-specific lure to ensure the maximum number of captures. Previous studies have isolated volatiles from the human skin microbiota and found the compound 3-methyl-1-butanol to be the most attractive to the malaria mosquito, *Anopheles gambiae*, in a laboratory setting; and recommended 3-methyl-1-butanol as a compound to increase *An. gambiae* captures in the field. To date, this compound’s ability to lure wild mosquitoes in differing land-use settings has not been tested. In this study, we evaluate the role of the synthetic compound, 3-methyl-1-butanol in combination with field produced CO^2^ in attracting *Anopheles* mosquitoes in varying land-use sites in Madagascar.

**Methods:**

CDC miniature light traps in combination with field produced CO^2^ were deployed in and around six villages near Ranomafana National Park, Madagascar. To test the role of 3-methyl-1-butanol in luring *Anopheles* mosquitoes, two traps were set in each land-use site (village, agricultural sites, and forested habitats affiliated with each village). One was baited with the synthetic odor and the other was kept as a non-baited control.

**Results:**

While 3-methyl-1-butanol baited traps did capture *An. gambiae s.l.* in this study, we did not find traps baited with synthetic 3-methyl-1-butanol to be more successful in capturing *Anopheles* mosquitoes, (including *Anopheles gambiae s.l.*) than the non odor-baited control traps in any of the land-use sites examined; however, regardless of odor bait, trapping near livestock pens resulted in the capture of significantly more *Anopheles* specimens.

**Conclusions:**

A strong synthetic lure in combination with insecticide has great potential as a mosquito control. Our findings suggest that trapping mosquitoes near livestock in malaria endemic regions, such as Madagascar, may be more successful at capturing *Anopheles* mosquitoes than the proposed 3-1-methyl-butanol lure.

## Background

Like many blood-feeding arthropods, mosquitoes in search of a blood meal use a suite of chemical cues to identify their host. This behavior is driven by a series of compounds, which are particularly attractive to mosquitoes [1]. In the case of one human malaria mosquito, *Anopheles gambiae*, compounds such as lactic acid and ammonia, which are found in the host’s skin microbiota, in combination with carbon dioxide are particularly attractive to host-seeking mosquitoes [2].

Skin microbiota composition varies greatly among humans making some individuals more attractive to host-seeking mosquitoes than others. Recent studies have revealed that this differential attraction to mosquitoes can be attributed to specific HLA gene regulated compounds that are produced on the human skin [3]. Olfactometer analysis of human skin emanations identified a series of volatiles produced by the bacteria living on the human foot, a location which mosquitoes are preferentially attracted to [2]. When cultured *in vitro*, the volatiles released by the bacteria were found to attract *A. gambiae* in laboratory-based olfactometer experiments.

To further test these compounds, Verhulst et al. [2,4] tested the effect of the ten compounds present in human foot bacteria on the host-seeking process of *A. gambiae* separately in an olfactometer in laboratory and semi-field conditions. Their findings [2] suggest that the compound 3-methyl-1-butanol is the most attractive compound of those produced by human foot bacteria to *A. gambiae*.

In this study we conducted field experiments to test the effect of 3-methyl-1-butanol as an odorant lure to *Anopheles* mosquitoes in differing land-use sites in and around Ranomafana National Park, Madagascar. One major difference between this study and the Verhulst et al. paper [4], is that this study did not use the 3-1-methyl-butanol in combination with a basic blend of ammonia, lactic and tetradecanoic acid, as the goal of this study was to examine the efficacy of 3-1-methyl-butanol as a lure on its own, an idea that was proposed in [4] as a potential collection tool. Additionally, the identification of a strong synthetic lure in combination with insecticides has exciting potential in the control of mosquito-vectors of disease,

The goal of this study was to examine the effectiveness of 3-methyl-butanol as an *Anopheles* lure in a malaria endemic region in the southeastern rainforests of Madagascar, where the rare endemic wildlife are threatened by habitat destruction, and malaria is a leading cause of mortality in humans.

## Methods

### Study site

The study site was in and around Ranomafana National Park (RNP) (21°02′–21°25′S, 47°18′–47°37′E), which is in the remaining southeastern rainforest of Madagascar. RNP is comprised of 43,500 hectares of continuous rainforest.

Adult mosquito trapping took place in eighteen sites in and around six villages bordering RNP. Forest sites had zero human inhabitants, and ranged from little (forest trails) to zero daily human overlap. Villages were defined as communities with at least 10 homes within 15 meters or less from one another. Three of the villages were within 1 km of slash-and-burn agricultural land use (tavy), and three were greater than 3 km from the nearest tavy agricultural site, but all six villages were within 3 km of RNP boundaries. Agricultural sites mainly consisted of rice paddies, but also included areas with vegetable gardens and banana trees.

### Mosquito trapping

Mosquitoes were collected from June through August 2013, using CDC miniature light traps (Model 512, John W. Hock Company, Gainesville, FL, USA) were active for 15 hours each evening. One of the two traps in each land-use site was also baited with a synthetic human-derived odor, 3-methyl-1-butanol (Fisher Scientific, Waltham, MA, USA catalog # 5001438080) [4]. A 6″ x 2″ piece of nylon hosiery was immersed into the concentrated odor and attached to the top of the light trap. Trapping at each location took place for 3-4 consecutive nights.

The odorant lure was used in combination with field-made CO^2^, and a control trap with the same field-made CO^2^ was set between 100 and 200 meters away from the baited trap. The CO^2^ was produced on-site through the fermentation in a 1.5L plastic bottles using 1 part yeast, 3 parts hot water gently mixed for 30 minutes, and then 1 part brown sugar was added (modified from [2]). One bottle of CO^2^ was made for each trap, and using rubber tubing, the CO^2^ output was streaming directly onto the CDC light trap.

There is also the potential that different light traps have different levels of attraction to mosquitoes. To avoid this potential discrepancy, trap locations, and odor-baited traps were randomized between every site.

Adult mosquitoes were identified morphologically to genus level, and those for which species identification was possible were also noted according to [5]. *Anopheles* mosquitoes were preserved in Drierite (Fisher Scientific, Waltham, MA, USA catalog #: 075783B) for further genetic and molecular analysis.

DNA was extracted from the abdomens of the collected female *Anopheles* mosquitoes for processing using the Collins protocol and heads and thoraces were kept for *Plasmodium* analysis; however, the molecular species identification results were inconclusive and hence this study relies on in-field morphological identifications, for which many individual mosquitoes were impossible to identify.

### Statistical analyses

A Poisson Regression model was performed to examine the relationship between the prevalence of Anopheles and independent variables in the study that may have affected their prevalence. The independent variables considered were: land-use, odor, village, moon illumination, temperature, precipitation and proximity to animal pens.

## Results and discussion

A total of 13,474 insects were captured. Of those, 2056 were mosquitoes of the genera *Culex, Mansonia,* and *Anopheles*, and 426 were *Anopheles* mosquitoes (Tables 1 and 2). The identifiable *Anopheles* species in this study were *An. gambiae s.l.*, *An. funestus*, *An. mascarensis*, *An. coustani*, *An. squamosus*, and several were identifiable as *Anopheles*, but species could not be determined. Of the *Anopheles gambiae s.l.* captured in this study 22.9%, 58.6%, and 0% were captured in the village, agricultural, and forest sites respectively, using the synthetic odor-bait (Table 1). Overall, there was no difference between the number of *Anopheles* mosquitoes captured using the synthetic odor and the non-odor controls (t(57) = .034, p = 0.97) (Table 3). When comparing odor and non-odor baited traps, fewer *Anopheles* mosquitoes were captured in the odor-baited (mean = 1.45, s.d. =2.58) traps than in the non-odor baited traps (mean = 4.3, s.d. = 6.84) in the village sites (t(19) = 2.1,p = 0.053); while the number of captured *Anopheles* mosquitoes did not differ between the odor-baited (mean = 8.4,s.d. = 13.72) and non-odor baited (mean = 5.25,s.d. = 11.53) traps in the agricultural sites (t(19) = 0.75, p = 0.46) and the forested sites (t(17) = 1.43, p = 0.17).

**Table 1:** **Mosquito species diversity captured in varying sites and habitat types in Ranomafana, Madagascar**

	**Ambatolahy**	**Vohiparara**	**Ambodiaviavy**	**Menarano**	**Manokoakora**	**Bevohazo**
Village	3 - *An.* s*quamosus*	6 - *M. uniformis*	0 - *Mansonia*	3 - *M. uniformis*	14 - *M. uniformis*	30 - *M. uniformis*
	6 (1) - *An.* g*ambiae*	3 (1) - *An.* f*unestus*	5 (2) - *An.* g*ambiae s.l.*	12 (6) - *An.* g*ambiae s.l.*	36 (9) - *An.* g*ambiae s.l.*	24 (1) - *An.* g*ambiae s.l.*
	4 - *An.* c*oustani*	1 – *An.* (unknown)	2 - *An.* s*quamosus*	3 (3) - *An.* f*unestus*	12 (6) - *An.* f*unestus*	3 - *An.* f*unestus*
	11 - C. *tritaenyorynchus*	107 - C. *quinquefasciatus*	2 - Culex (unknown)	1 - *Anopheles* (unknown)	5 - *Anopheles* (unknown)	2 - *An.* m*ascarensis*
	7 - C. *quinquefasciatus*	36 - *Culex* (unknown)	7 - C. *quinquefasciatus*	3 - C. *quinquefasciatus*	11 - C. *quinquefasciatus*	2 - *Anopheles* (unknown)
	0 - *Mansonia*	4 - C. *tritaenyorynchus*	4 - C. *tritaenyorynchus*		4 - *Culex* (unknown	36 - C. *quinquefasciatus*
						24 - C. *tritaenyorynchus*
						3 - C. *decens*
						32 - *Culex* (unknown)
Agricultural	3 (2) - *An.* g*ambiae s.l.*	3 - M. *uniformis*	0 - *Mansonia*	56 (5) - *An.* g*ambiae s.l.*	12 - M. *uniformis*	7 - M. *uniformis*
	2 (2) - *An.* c*oustani*	1 (1) - *An. mascarensis*	40 (26) - *An. gambiae s.l.*	19 - *An.* f*unestus*	67 (63) - An. g*ambiae s.l.*	6 (5) - *An.* f*unestus*
	1 (1) - *An.* m*ascarensis*	2 (2) - *Anopheles* (unknown)	24 (19) - *An. funestus*	3 - An. m*ascarensis*	9 (9)- *An. mascarensis*	3 (3) - *An.* g*ambiae s.l.*
	10 - C. *tritaenyorynchus*	12 - C. *tritaenyorynchus*	1 - *An. mascarensis*	5 - *Anopheles* (unknown)	12 (12) - An. f*unestus*	26 - Culex (unknown)
	9 - C. *quinquefasciatus*	39 - C. *quinquefasciatus*	23 - C. *quinquefasciatus*	10 - C. *quinquefasciatus*	18 (18) - *Anopheles* (unknown)	14 - C. *quinquefasciatus*
	0 - *Mansonia*	12 - C. *antennatus*	2 - C. *tritaenyorynchus*	2 - *Culex* (unknown)	77 - C. *quinquefasciatus*	1 - C. *decens*
		4 – *Culex* (unknown)	9 - *Culex* (unknown)		2 - C. *tritaenyorynchus*	1 - C. *antennatus*
			3 - C. *antennatus*		16 - C. *decens*	1 - C. *tritaenyorynchus*
					16 - *Culex* (unknown)	
Forest	0 - *Anopheles*	5 - M. *uniformis*	0 - *Mansonia*	1 - M. *uniformis*	35 - M. *uniformis*	58 - M. *uniformis*
	12 - C. *tritaenyorynchus*	45 - C. *quinquefasciatus*	1 (1) - *An.* f*unestus*	3 - An. c*oustani*	11 (2) - *Anopheles* (unknown)	277 - C. *quinquefasciatus*
	11 - C. *quinquefasciatus*	3 - C. *tritaenyorynchus*	*37 - C. quinquefasciatus*	45 - C. *quinquefasciatus*	3 - An. g*ambiae s.l.*	31 - C. *antennatus*
	1 - C. *decens*	27 - C. *antennatus*	4 - *Culex* (unknown)	2 - C. *antennatus*	101 - C. *quinquefasciatus*	63 - C. *tritaenyorynchus*
	0 - *Mansonia*	24 - *Culex* (unknown)		6 - C. *decens*	48 - C. *antennatus*	2 - C. *decens*
		0 - *Anopheles*		13 - Culex (unknown)	17 - C. *tritaenyorynchus*	57 - Culex (unknown)
				1 - C. *tritaenyorynchus*	28 - *Culex* (unknown)	0 - Anopheles
				2 - C. *poicilipes*		

Abbreviations are as follows: *An.* for *Anopheles, M.* for *Mansonia,* and *C.* for *Culex* species. The total number of species is listed for each site.

The number of mosquitoes captured using a synthetic odorbaited trap are indicated in parenthesis.

**Table 2:** **Total number of mosquitoes and percentage of** ***Anopheles*** **mosquitoes collected in forest, agricultural, and village sites in and around Ranomafana national park, Madagascar**

**Site**	**Total #**	**Total # mosquitoes**	**Total #**
	**mosquitoes forest**	**agriculture**	**mosquitoes village**
	**(%** ***Anopheles*** **)**	**(%** ***Anopheles*** **)**	**(%** ***Anopheles*** **)**
Ambatolahy	24 (0%)	25 (24%)	35 (37%)
Ambodiaviavy	42 (2.4%)	102 (63.7%)	20 (35%)
Bevohazo	488 (0%)	86 (10.5%)	156 (19.9%)
Manokoakora	243 (5.8%)	229 (46.3%)	82 (64.6%)
Menarano	73 (4.1%)	95 (87.4%)	22 (72.7%)
Vohiparara	104 (0%)	73 (4.1%)	157 (2.5%)

**Table 3:** **Total number of** ***Anopheles*** **mosquitoes captured using odor and non-odor baited traps and the proportion of those that were blood-fed in and around six villages near Ranomafana national park, Madagascar**

**Site**	**Average temperature (Celsius) (SE)**	**Odor**	**Non-odor**	**Total**	**Percentage blood-fed (O/N) odor/non-odor**
Ambatolahy	11.13 (0.07)	6	25	31	16.6% (N)
Vohiparara	13.2 (0.08)	4	3	7	0
Ambodiaviavy	11.39 (0.06)	48	25	73	0
Menarano	10.56 (0.09)	14	88	102	12.7% (O)
Manoakokora	12.6 (0.06)	119	54	173	0
Bevohazo	11.26 (0.03)	9	31	40	0

**Figure 1:**
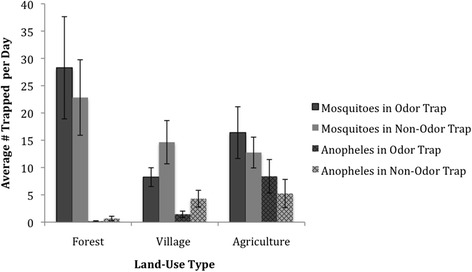
**Average number of mosquitoes/** ***Anopheles*** **trapped per night by land-use type in and around Ranomafana national park, Madagascar, with standard error bars, stratified by odor/non-odor trap (n = 6).**

The total number of mosquitoes captured did not differ between odor and non-odor baited traps (t(57) = 0.28,p = 0.77) (Figure 1). There was also no difference in the number of mosquitoes captured using non-odor baited (mean = 14.65,s.d. = 17.72), and odor baited traps (mean = 8.25,s.d. = 7.70) in the village sites (t(19) = 1.90,p = 0.073), agricultural sites (t(18) = 0.55, p = 0.59), and forested sites (t(17) = 1.05, p = 0.31). Odor was a statistically significant independent variable in a Poisson model with the ratio of *Anopheles* to total mosquitoes as the dependent variable in that the non-odor traps have a log count 0.666 higher than the odor traps, meaning non-odor traps had a higher percentage of *Anopheles* out of total mosquitoes.

A full model was run with all seven independent variables. The following variables were significant: land-use (p-value: <0.0001), village (p-value: <0.0001), odor (p-value: <0.0001) and proximity to animals (p-value: <0.0001). When the full model was performed to examine the relationship between the ratio of the number of Anopheles out of the total number of mosquitoes and all 7 of the independent variables listed above, the following variables were significant: land-use (p = <0.0001), village (p = <0.0001), proximity to animals (p = <0.0001) and moon illumination (p = 0.0047).

Since one trap at each land-use site was baited with an odor (3-methyl-1-butanol) and the other was not. Odor was not included in the final Poisson Regression model with *Anopheles* as the dependent variable, because the full model did not show a statistical significance in the trapping numbers between the odor and non-odor baits (p-value = 0.55). However, odor was a statistically significant independent variable in the Poisson final model with the ratio of *Anopheles* to total mosquitoes as the dependent variable.

While morphological identification was conducted in the field, we were unable to identify all *Anopheles* mosquito species upon return. Heads and thoraces were used for *Plasmodium* ELISA testing, and inconclusive molecular results were obtained while attempting to identify mosquito species molecularly.

We found no evidence that the odor lure on odor-baited traps lost its potency over the four-day trapping period in each site. The number of mosquitoes collected in odor-baited traps was consistent across consecutive days of trapping.

Odor-baited traps did not attract more blood-fed *Anopheles* mosquitoes than our control traps. However, even with such low capture rates our trapping methods were successful in capturing blood-fed (over 4% of *Anopheles* and 8% of *Culex*) mosquitoes. One potential explanation for this may be that the use of field made CO^2^ at both odor-baited and control traps increased attraction of bloodfed mosquitoes even in the absence of the synthetic odor bait [6].

While we did find evidence that the CDC light traps located near human dwellings and animal pens were more likely to capture *Anopheles* mosquitoes, this may not only be due to odour-mediated preferences of zoophilic or anthropophilic *Anopheles* [7], but also due to *Anopheles* abundance (as locations in and around villages may be more ideal *Anopheles* breeding habitats). The aim of this study was not to directly investigate the attractiveness of livestock and human odors to mosquitoes; however we did find evidence that CDC light traps located in close proximity to livestock pens do capture significantly more *Anopheles* mosquitoes. This information may be a useful tool in future *Anopheles* surveillance studies in the region.

While odor and temperature did not appear to influence mosquito capture numbers, n this study lunar phase influenced the total number of insect captures, total mosquito captures, and *Anopheles* captures consistently across all traps. A new moon resulted in the highest number of total insect captures (n = 950) in our CDC mini light traps, while a full moon resulted in the lowest number of total insect captures (n = 256). During the new moon, we captured 42 mosquitoes, 19 (45.2%) of which were *Anopheles*. In contrast, during the full moon, 81 mosquitoes were captured, 73 (90.12%) of which were *Anopheles*. Further work, collecting microhabitat information on the trap locations may help to better elucidate the role that lunar phase, temperature, humidity, and land-use type may play. Hourly collections coinciding with these variables may reveal peak capture conditions.

Excluding differences in odor vs. non-odor baited traps, we captured more mosquitoes and more *Anopheles spp.* mosquitoes in close proximity to animal pens. When odor data were stratified by proximity to an animal pen, close proximity to animal pens appears to increase *Anopheles* numbers more than odor traps, even in agricultural sites. Although this study was not designed to look at animal influence on *Anopheles* prevalence, once traps were randomly placed within a land-use site, the proximity to animal pens was noted. The relationship of *Anopheles* prevalence and proximity to an animal pen was investigated using Poisson regression analysis. In the model with *Anopheles* as the dependent variable, traps that were not located in close proximity to animal pens had a log count 1.8122 lower (p-value- < 0.0001) than that of the traps located in close proximity to animal pens. When looking at the ratio of *Anopheles* of total mosquitoes, traps that were not located in close proximity to animal pens had a log count 0.5142 lower (p-value- < 0.0001) than that of the traps located in close proximity to animal pens.

In this study, we were unable to distinguish *A. arabiensis* and *A. gambiae*; however, our morphological identification revealed evidence of *An. mascarensis* and *An. squamosus* in agricultural and village sites. Since these species are often considered zoophilic [7], they may not have been attracted to the bait used in this study, which was derived from human odor, which may explain why we did not detect evidence that the 3-methyl-1-butanol odor lure was more attractive to *Anopheles* mosquitoes than a non odor baited lure. Similarly, *An. funestus* in this study was captured primarily in village and agricultural sites; as this is known as an anthropophilic species, perhaps evidence of *An. funestus* in odor-baited sites would have supported [4] the notion that 3-methyl-1 butanol is an effective lure for anthropophilic *Anopheles* mosquitoes. .

Previous studies have found that A*. arabiensis* show a high degree of zoophily in Madagascar [8,9], perhaps due to the 2:1 ratio of cattle to humans on the island (http://www.fao.org/ag/agp/AGPC/doc/Counprof/Madagascar/madagascareng.htm#4.RUMINANT). Therefore, *A. arabiensis* populations in Madagascar may not be innately zoophilic, but rather express zoophily in response to the environmental conditions produced by agricultural practices, deforestation, and land-use change in Madagascar. For example, as shown in [7] *An. arabiensis* and *An. gambiae* have an innate preference for calf odor over human odor in Madagascar. Further studies investigating the vector potential of captured *Anopheles* and screening bloodfed *Anopheles* to identify hosts will further elucidate this dynamic. In this study, we found that placing traps in close proximity to livestock pens (where large bovids, zebu, are housed) significantly increased the number of *Anopheles* mosquitoes captures when compared to 3-methyl-1-butanol baited traps.

## Conclusions

While 3-methyl-1-butanol has been found to be the most attractive compound to malaria mosquitoes in laboratory studies [4], we did not find the compound alone to be better at capturing *Anopheles* mosquitoes in field settings in Madagascar. However, we did find that the location of the trap influenced the number of *Anopheles* mosquitoes captured more than the odor-bait, with nearly three times as many individuals captured in traps in close proximity to livestock pens.
